# A feather cyst causing vertebral bone lysis and spinal cord compression in a Lohmann Brown layer

**DOI:** 10.4102/jsava.v91i0.1758

**Published:** 2020-02-06

**Authors:** Alaster Samkange, Borden Mushonga, Erick Kandiwa, Alec S. Bishi, Basiamisi V.E. Segwagwe, Erdwin Muradzikwa

**Affiliations:** 1 Department of Production Animal Clinical Studies, School of Veterinary Medicine, University of Namibia, Windhoek, Namibia; 2 School of Veterinary Medicine, University of Namibia, Windhoek, Namibia; 3 Department of Population Health, School of Veterinary Medicine, University of Namibia, Windhoek, Namibia; 4 Department of Biomedical Sciences, Faculty of Medicine, University of Botswana, Gaborone, Botswana; 5 Department of Production Animal Health, School of Veterinary Medicine, University of Namibia, Windhoek, Namibia

**Keywords:** Lohmann Brown layer, spinal cord compression, feather cyst, Neudamm, Namibia

## Abstract

A feather cyst in the cervical region which, through complications of inward growth, resulted in compression of the cervical spinal cord of a Lohmann Brown layer is described. It is postulated that expansion of the cyst exerted pressure on the adjacent cervical vertebra and through bone lysis created an opening through which the cyst protruded, thereby exerting pressure on the spinal cord. The affected spinal cord segment was reduced to a fifth of its normal diameter. The bird most likely died of starvation because of limb and neck paralysis and disorientation. Although the cause of the feather cyst was not conclusively identified, moulting and trauma could have triggered its formation and subsequent growth.

## Introduction

A feather follicle cyst, also known as *hypopteronosis cystica* or *pterylofolliculosis cystica* or plumafolliculoma (Reece 1996; Zwart & Grimm 2003), is a benign (Pass 1989) skin condition that occurs when a developing feather fails to break through and emerge out of the epidermis, resulting in the feather growing inside the cyst (Olszewski 1987). A feather cyst usually develops when feathers of a young bird come out for the first time or when they are replaced by new feathers or at the time of moulting (Zwart & Grimm 2003).

Feather follicle cysts have been associated with a number of pathological conditions including dysplasia of the developing feather follicle, benign neoplasia, malnutrition and skin injuries that interfere with the feather’s ability to break through the skin (Pass 1989; Zwart & Grimm 2003). In addition, feather cysts have been linked to quill mite (*Dermoglyphus passerinus*) infestations (Dorrestein et al. 1997), as well as bacterial, fungal and viral infections (Bernier, Morin & Marselais 1984; Davidson et al. 1989; Zwart & Grimm 2003).

Feather follicle cysts are rare in domestic fowl, commonly occurring in small wild and cage birds (Mutinelli et al. 2008; Reece 1996; Rosskopf 2003). Feather cysts have only been described once in chickens (Mutinelli et al. 2008). They have also been described in a wild turkey (*Meleagris gallopavo*) (Couvillion, Maslin & Montgomery 1990) and in a barn owl (*Tyto alba*) (Frasca et al. 1999).

According to some authors, feather cysts can be dry or wet (Pass 1989), single or multiple, soft or hard, closed or open, fixed or sessile skin nodules with variable diameters (1 cm – 4 cm) with rather thin walls (Zwart & Grimm 2003). The contents can also be doughy, soft and crumbly concentric lamellar structures with a formed feather in the middle (Couvillion et al. 1990; Frasca et al. 1999; Zwart & Grimm 2003).

The current report describes the gross lesions, proposed pathogenesis and complications of a feather cyst that led to the death of a Lohman Brown layer.

## Patient presentation

University of Namibia’s Neudamm Farm near Windhoek utilises about 1000 point-of-lay Lohmann Brown layers imported from South Africa, primarily for the purpose of student training and research. The birds are housed in battery cages and are fed standard commercial layer’s feed (mash) purchased from a local animal feed manufacturer. After one year of laying, the birds are disposed of and are replaced by a new flock after routine decontamination of the premises.

A 73-week-old Lohmann Brown layer chicken was presented for post-mortem examination. The bird had a history of paresis, ataxia and disorientation before it was found dead. The rest of the bird’s battery cage mates were apparently healthy with no clinical signs of disease observed.

A necropsy was conducted according to the method described previously (Bello et al. 2012; Nyaga et al. 2014). An external examination of the carcass was followed by de-feathering of the ventral abdomen, thorax and thighs before the bird was put in dorsal recumbency. The whole carcass, including the feathered back and wings, was visually examined for parasites using a hand lens and thoroughly palpated.

## Pathological examination

On external examination, the chicken was found to be in a poor condition, with an estimated body condition score of 1 on a scale of 0–3 (Gregory & Robins 1998). Examination of the plumage revealed that it had been recovering from a recent moult. Few isolated to coalescing patches of alopecia over the back, cervico-thoracic junction and wings were noticeable. The skin was shrunken and the eyes were sunken.

**FIGURE 1:**
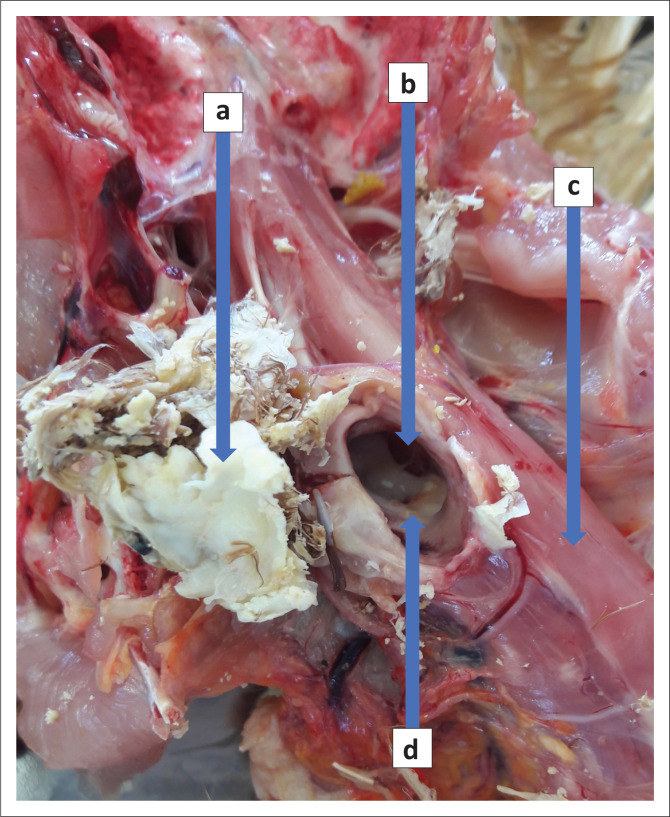
The feather cyst (a) after removal from a foramen (b) through the cervical vertebrae (c) causing compression of the cervical spinal cord (d).

**FIGURE 2:**
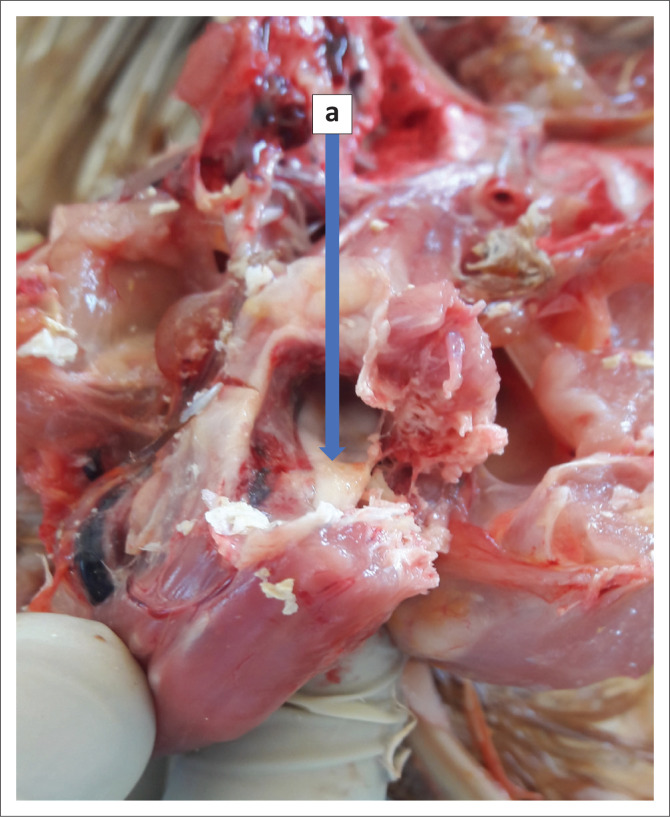
Cervical spinal cord compression (a) clearly visible through the cut surface of the cervical vertebra.

**FIGURE 3:**
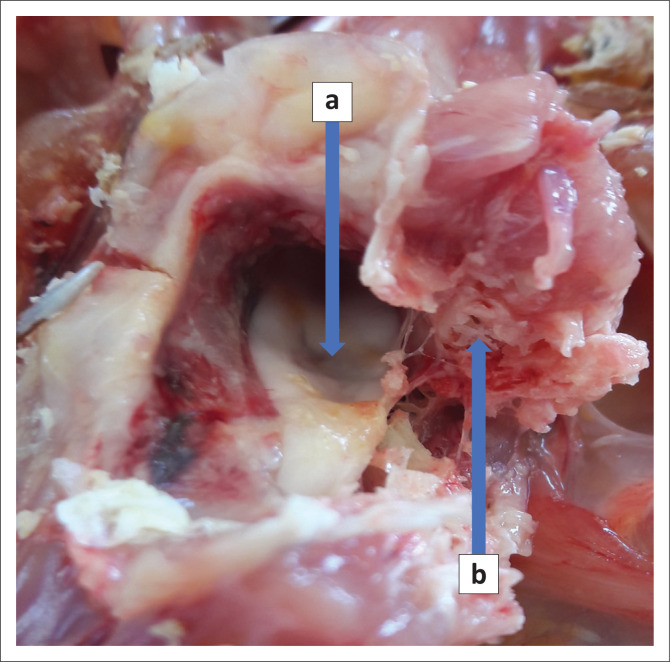
Close-up of the cervical spinal cord compression (a) caused by the feather cyst as seen through the foramen caused by bone lysis and the sectioned cervical vertebra (b).

**FIGURE 4:**
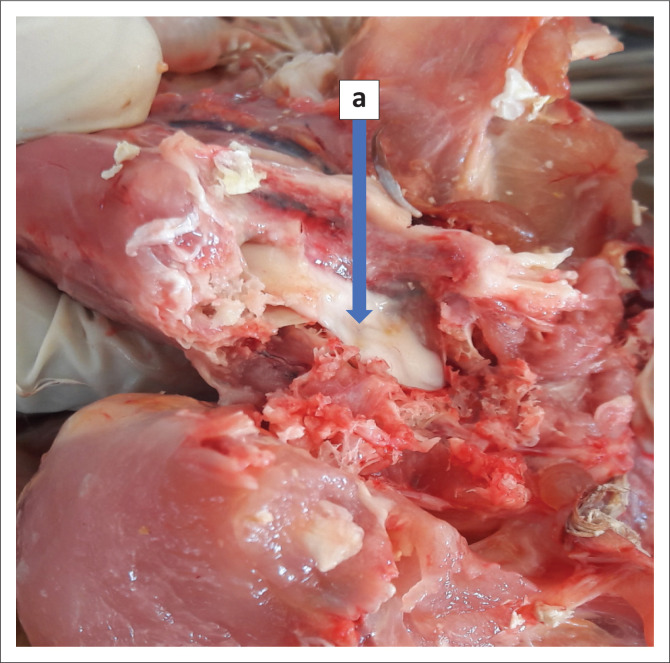
The exposed spinal cord with a clearly visible compression (a) lesion caused by the cyst.

**FIGURE 5:**
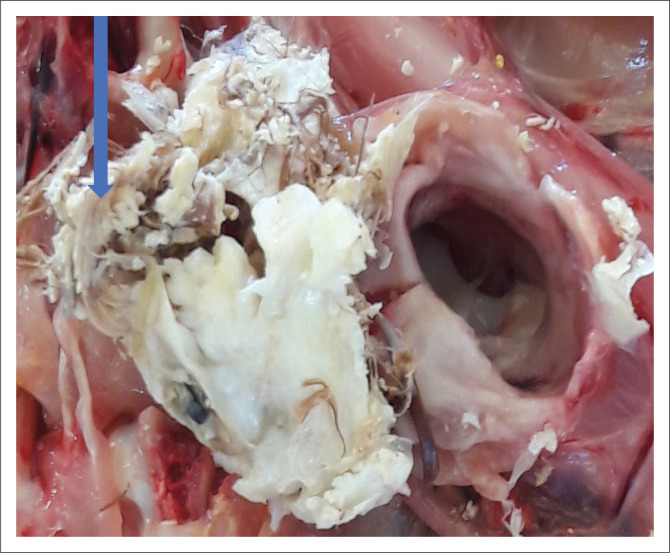
A close-up picture clearly depicting a feather within the cyst contents as shown by the arrow.

A single ovoid cystic nodule was found on the caudal neck area at the cervico-thoracic junction. The nodule was firm, irregular to oval in shape, 20 mm long × 15 mm wide and about 10 mm deep. It was located on the right dorso-lateral aspect of the neck over the last two cervical vertebrae. It extended from the skin surface through the subcutaneous tissue and muscle, all the way to the cervical vertebral column. Removal of the cyst from the neck revealed a large opening of about 7 mm diameter, extending from the dorsal portions of the bodies to the laminae of the last two cervical vertebrae (Figure 1). The bodies of the vertebrae were only slightly lysed. A portion of the cyst was protruding through the opening into the spinal canal and caused pronounced compression and flattening of the spinal cord. Subsequent dissection of the spinal cord revealed that it was reduced to approximately 20% of its diameter for a distance of 6 mm (Figures 2–4). Upon opening of the cyst, it contained a yellowish white, calcareous, keratinised, concentric laminated and semisolid substance surrounding parts of a partially developed feather (Figure 5). The crumbly yellowish white material was easily broken down and washed away by using a gentle tap water jet from the sink thereby exposing a feather rachis and its two vanes.

The muscles were pale and the keel was prominent, appearing like a knife blade because of atrophy of the pectoral muscles. Involvement of the musculoskeletal system was limited to the area of the lesion. The nervous system appeared normal except for a part of the spinal cord around the last two cervical vertebrae that were overlain by the cystic lesion. The crop and gizzard were empty. There was a moderate amount of transudate in the trachea whilst the lungs were bright pink and produced a frothy fluid upon transection. The ovary was shrunken and had very small follicles; the oviduct was also very small.

On the basis of these necropsy findings, a diagnosis of death because of starvation secondary to cervical spinal cord compression by a feather cyst was established.

## Discussion

The Lohmann Brown breed was initially developed from the Leghorn in the United States of America and later from the New Hampshire in Germany through selective breeding (Countryside 2018). The German stock are the parents of the South African Lohmann Brown breed that provided parentage to the stock used in the current study (Lohmann Tierzucht 2018). Although it was previously suggested that Lohmann Brown layers are susceptible to feather problems (Singh et al. 2009), feather follicle cysts were not specifically mentioned. Furthermore, complications of feather cyst lesions have not been reported to result in death of domestic fowl species in previous studies except in a case in which fatality was a direct result of blood loss when surgical excision was attempted (Pass 1989).

The causes of feather cysts cannot be easily identified in each case but can, in general, be categorised into inherited and acquired causes that include trauma, aplasia, neoplasia, parasitic infestation, fungal, bacterial and viral infection, or conditions that interfere with the normal growth of feathers (Schmidt & Lightfoot 2006). Hypersensitivity reactions, endocrinopathy and nutritional deficiency have also been mentioned as possible underlying causes (Nett & Tully 2003). Differential diagnoses for this condition include neoplasia (plumafolliculoma), trauma (Reece 1996; Zwart & Grimm 2003), infection with bacteria, viruses (Bernier et al. 1984) and fungi (Davidson et al. 1989) and infestation with parasites (Dorrestein et al. 1997). It has also been suggested that feather cysts may arise from a combination of the above causes (Pass 1989; Schmidt & Lightfoot 2006). In addition, the risk of feather cyst development increases with the number of moults (AvianWeb 2018). We propose that the feather cyst reported in this study could have resulted from a feather that failed to find its way through the skin as a result of skin injury, because of either feather pecking or cage fence trauma during the preceding moult, as alluded to by some authors (Zwart & Grimm 2003).

In conclusion, the authors propose that the described feather cyst by extension caused pressure atrophy and localised lysis of an adjacent vertebra, thereby forming an opening in the vertebral lamina through which the cyst protruded and resulted in compression of the spinal cord. The spinal cord lesion most likely caused progressive limb and neck paresis, inability to feed and eventual starvation and death.

## References

[CIT0001] AvianWeb, 2018, *Feather cysts/feather lump*, viewed 22 July 2018, from https://www.beautyofbirds.com/feathercysts.html.

[CIT0002] Bello, A., Umaru, M.A., Baraya, Y.S., Adamu, Y.A., Jibir, M., Garba, S. et al., 2012, ‘Postmortem procedure and diagnostic avian pathology’, *Scientific Journal of Zoology* 1(3), 1–5.

[CIT0003] Bernier, G., Morin, M. & Marsolais, G., 1984, ‘Papovavirus induced feather abnormalities and skin lesions in the budgerigar: Clinical and pathological findings’, *Canadian Veterinary Journal* 25(8), 307–310. PMC1790627 17422435

[CIT0004] Countryside, 2018, ‘The history of Rhode Island red chickens’, *Countryside Daily*, viewed 29 July 2018, from https://countrysidenetwork.com/daily/poultry/chickens-101/history-rhode-island-red-chickens/.

[CIT0005] Couvillion, C.E., Maslin, W.A. & Montgomery, R.M., 1990, ‘Feather follicle cysts in a wild turkey’, *Journal of Wildlife Diseases* 26(1), 122–124. 10.7589/0090-3558-26.1.122 2304193

[CIT0006] Davidson, W.R., Shotts, E.B., Teska, J. & Moreland, D.W., 1989, ‘Infections’, *Journal of Wildlife Diseases* 25(4), 534–539. 10.7589/0090-3558-25.4.534 2810554

[CIT0007] Dorrestein, G.M., Van Der Horst, H.H.A., Cremers, H.J.W.M. & Van Der Hage, M., 1997, ‘Quill mite (*Dermoglyphus passerinus*) infestation of canaries (*Serinus canaria*): Diagnosis and treatment’, *Avian Pathology* 26(1), 195–199. 10.1080/03079459708419205 18483901

[CIT0008] Frasca, S. Jr., Schwartz, D.R., Moiseff, A. & French, R.A., 1999, ‘Feather folliculoma in a captive-bred barn owl (*Tyto alba*)’, *Avian Diseases* 43(3), 616–621. 10.2307/1592666 10494437

[CIT0009] Gregory, N.G. & Robins, J.K., 1998, ‘A body condition scoring system for layer hens’, *New Zealand Journal of Agricultural Research* 41(4), 555–559. 10.1080/00288233.1998.9513338

[CIT0010] Lohmann Tierzucht, 2018, *Layers, Lohmann Tierzucht*, viewed 29 July 2018, from http://www.ltz.de/en/layers/index.php?navid=608984608984.

[CIT0011] Mutinelli, F., Corro, M., Catania, S. & Melchiotti, E., 2008, ‘Multiple feather follicle cysts in a Moroseta hen (*Gallus gallus*)’, *Avian Diseases* 52(2), 345–347. 10.1637/8153-101907-Case.1 18646468

[CIT0012] Nett, C.S. & Tully, Jr. T.N., 2003, ‘Anatomy, clinical presentation, and diagnostic approach to feather-picking pet birds’, *Compendium on Continuing Education for the Practicing Veterinarian* 25(3), 206–219.

[CIT0013] Nyaga, P.N., Bebora, L.C., Mbuthia, P.G., Njagi, L.W. & Gathumbi, P.W., 2014, ‘Diagnostic poultry post-mortem examination in avian medicine’, paper presented at the Faculty of Veterinary Medicine Poultry Workshop, Department of Veterinary Pathology, Microbiology and Parasitology, University of Nairobi, Nairobi, viewed 18 June 2018, from https://vetpathology.uonbi.ac.ke/.

[CIT0014] Olszewski, A.B., 1987, ‘Mutations and hereditary disorders’, in E.W. Burr (ed.), *Companion bird medicine*, pp. 33–35, Iowa State University Press, Ames, IA.

[CIT0015] Pass, D.A., 1989, ‘The pathology of the avian integument: A review’, *Avian Pathology* 18(1), 1–72. 10.1080/03079458908418580 18679837

[CIT0016] Reece, R.L., 1996, ‘Some observations on naturally occurring neoplasms of domestic fowls in the State of Victoria, Australia (1977–87)’, *Avian Pathology* 25(3), 407–447. 10.1080/03079459608419153 18645870

[CIT0017] Rosskopf, W.J., 2003, ‘Common conditions and syndromes of canaries, finches, lories and lorikeets, lovebirds, and macaws’, *Seminars in Avian and Exotic Pet Medicine* 12(3), 131–133. 10.1053/seap.2003.00023-9

[CIT0018] Schmidt, R.E. & Lightfoot, T.L., 2006, ‘Integument’, in G.J. Harrison & T.L. Lightfoot (eds.), *Clinical avian medicine – volume 1*, pp. 395–409, Spix Publishing, Inc., Palm Beach, FL.

[CIT0019] Singh, R., Cook, N., Cheng, K.M. & Silversides, F.G., 2009, ‘Invasive and noninvasive measurement of stress in laying hens kept in conventional cages and in floor pens’, *Poultry Science* 88(7), 1346–1351. 10.3382/ps.2008-00300 19531702

[CIT0020] Zwart, P. & Grimm, F., 2003, ‘Plumafolliculoma, (feather cyst), in canary birds, a benign tumor’, paper presented at the 7th Meeting of Association of Avian Veterinarians, Loro Parque, Tenerife, viewed 20 September 2018, from https://www.researchgate.net/.

